# Involvement of African patient populations in clinical trials on leprosy: a scoping review

**DOI:** 10.1186/s41182-026-00913-x

**Published:** 2026-04-03

**Authors:** Marie Haarmann, Ruth Ansah, Augusto Meneguim, Michael Ramharter, Johannes Mischlinger, Thomas Baranek, Mirjam Groger

**Affiliations:** 1 Center for Tropical Medicine, Bernhard Nocht Institute for Tropical Medicine & I Dept. of Medicine University Medical Center Hamburg-Eppendorf, Hamburg, Germany; 2 Cape Coast Teaching Hospital, Cape Coast, Ghana; 3 Department of Clinical Research, University of Applied Sciences and Arts, Hannover, Germany; 4 Centre de Recherches Médicales de Lambaréné (CERMEL), Lambaréné, Gabon; 5 German Center for Infection Research, Partner Site Hamburg-Lübeck-Borstel-Riems, Hamburg, Germany

**Keywords:** Leprosy, Infectious diseases, Africa, *Mycobacterium leprae*, Scoping review, Treatment trials, Prophylaxis trials, Vaccine trials

## Abstract

**Background:**

Leprosy is a neglected tropical disease of public health importance. Although Africa carries a substantial share of the global leprosy burden, there is only limited evidence for African patients based on leprosy-related clinical trials. This scoping review aims to map existing evidence on the involvement of African patient populations in leprosy-related clinical treatment, prophylaxis and vaccine trials.

**Methods:**

A scoping review was performed in 2023 by two independent reviewers following the PRISMA guideline. The electronic databases PubMed and Infolep, and clinical trial registries (Cochrane CENTRAL, WHO International Clinical Trials Registry Platform) were systematically searched for past and ongoing clinical trials on leprosy with recruitment in Africa up to 31 December 2022. 16 trials were registered on the WHO platform, but none had published results. Predefined data points were extracted, and study quality was assessed using Cochrane’s RoB2 and ROBINS-I tools. The review is registered in PROSPERO.

**Results:**

Out of 198 publications, 22 were eligible for extraction, representing 18 clinical trials. The majority of trials were conducted in Malawi, Ethiopia and Uganda. 12 trials focused on treatment, 2 on vaccines, 3 on treatment reactions and 1 on prophylaxis. 10 clinical trials were randomized, 14 were controlled and 6 trials were blinded. One of these trials was pseudo-randomized and, therefore, not considered as randomized. Dapsone was the most frequently studied drug. 15 (83%) of all identified studies were conducted before the year 2000 having one study that published one paper before 2000 and one after 2000. 11 studies were assessed with the RoB2 tool and 8 (73%) showed high risk of bias. One study with two publications showed one serious and one medium risk of bias. Among 7 studies assessed with the ROBINS-I tool, 2 (29%) showed a serious risk of bias.

**Conclusions:**

This scoping review demonstrates the substantial under-representation of the African patient population in leprosy clinical trials and highlights the low volume and a decrease in clinical trial conduct since the year 2000. To address this imbalance and improve the relevance of trial outcomes, there is a critical need to engage local stakeholders and build research capacities in Africa. These efforts will be essential for more inclusive and effective leprosy interventions.

**Supplementary Information:**

The online version contains supplementary material available at 10.1186/s41182-026-00913-x.

## Background

Leprosy or Hansen’s disease is an infectious disease caused by *Mycobacterium leprae* and *Mycobacterium lepromatosis*. It primarily affects the skin and peripheral nerves [1]. Although leprosy is curable, it has a major impact on those affected when not treated early on [1]. Late treatment can result in permanent sequelae, leading to disabilities and social exclusion with accompanying poor mental well-being and quality of life [2].

Leprosy has been shown to be associated with two distinct forms of reaction [3]. The type 1 reaction (T1R) is characterized by an initially weakened immune response that subsequently intensifies. Furthermore, neuritis has been observed to be associated with T1R, which can result in nerve enlargement, tenderness, and loss of function. The type 2 reaction known as "*erythema nodosum leprosum* (ENL)" is a rare immune-mediated condition that can arise from various underlying causes, including, but not limited to, tuberculosis, sarcoidosis, Crohn's disease, or an adverse reaction to certain medications [4, 5].

Despite global advances in disease control, leprosy remains a public health concern in several low- and middle-income countries. The African continent contributes substantially to the global leprosy burden, with thousands of new cases reported annually, particularly in higher endemic regions, such as Ethiopia, Nigeria, the Democratic Republic of Congo, and Mozambique. However, global leprosy research trends indicate that only few scientific publications come from African countries and African institutions, while countries from other continents, such as Brazil, India or the United States of America have the highest research outputs in the field of leprosy [6].

Clinical research has played a relevant role in improving global leprosy control. When dapsone monotherapy became increasingly ineffective, the multi drug therapy (MDT) recommended by the World Health Organisation (WHO) in 1981 was introduced. Most clinical trials included in this review were published after this recommendation and contributed to confirming safety and efficacy. Therefore, equitable representation of affected populations in these trials is important to ensure the applicability of findings to respective patient populations.

In this context, understanding the extent and nature of African patient involvement in leprosy clinical trials is essential for identifying research gaps and addressing systemic barriers to inclusion. Therefore, this scoping review sought to explore the current landscape of leprosy clinical trials involving African populations, with the aim of guiding future research priorities and strengthening Africa’s contribution to the global leprosy research agenda.

## Methods

This review was registered at PROSPERO (CRD42023437175). The electronic resources PubMed and Infolep were systematically searched with the search terms detailed in Supplementary file 1. References were screened for additional publications. Furthermore, the Cochrane Controlled Registry of Trials (CENTRAL) and the WHO International Clinical Trials Registry Platform, including the Pan African Clinical Trial Registry, were screened for registrations of additional clinical trials. The search terms are also detailed in Supplementary file 1. The electronic sources for this scoping review were selected to provide comprehensive coverage of biomedical literature, leprosy-specific research, and registered clinical trials: PubMed and Cochrane CENTRAL are widely recognized for indexing high-quality clinical and biomedical studies, while Infolep is a specialized resource for leprosy-related publications, including those not indexed in PubMed. The WHO ICTRP ensures inclusion of ongoing and completed trials globally.

The review was conducted in 2023 and includes all publications up until 31st December 2022. A PRISMA checklist for this manuscript can be found in Supplementary file 2.

### Eligibility criteria

To be eligible, publications had to meet the following criteria: (a) the presented study population had to be at risk of leprosy, i.e., participants had to live in a country, where leprosy was endemic and would, therefore, be eligible for inclusion in vaccination and prophylaxis trials, or they had to be diagnosed with leprosy for inclusion to a treatment trial. In addition, (b) in case of multicenter multi-country trials, at least one of the recruiting study centers presented in the publication had to be in an African country. Inclusion of ‘Africa’ was based on geographic location, encompassing all nations located within the African continent. Finally, (c) the trial had to be an interventional clinical trial evaluating an investigational medicinal product (IMP) for prophylaxis, treatment, or vaccination. No language restrictions were applied. In addition to the search terms, appropriate filters were applied to restrict the results to clinical trials. Results up to 31. December 2022 were included in this scoping review.

### Study selection and data extraction

Records were imported into the reference management software EndNote (version 9, Clarivate Analytics) and duplicates were removed using the automated de-duplication tool. In addition to the automated duplicate removal tool, manual screening and removal of duplicate records were performed. The search results were first manually screened by title and abstract, based on the eligibility criteria. For eligible records, full texts were obtained and screened. Reasons for exclusion were documented in Supplementary file 3 as applicable to ensure transparency throughout the selection process. Manual search of references for relevant content that had not been part of the initial search was done in addition. From the final set of eligible records, the following information was extracted and documented in a standardized Excel form (version 2405, Microsoft 365): author, year of publication, period of study conduct, type of trial, randomization, trial arms with control group(s), blinding, recruiting countries, objective, primary endpoints, reached sample size, population, confirmation of diagnosis, sex, age range, age median/mean. Two independent reviewers worked through the screening, selection and extraction process. Discrepancies were resolved through discussion or consultation with a third reviewer. The review was conducted by two reviewers with complementary backgrounds. One reviewer has a medical background and contributed clinical understanding and subject-matter expertise. The second reviewer has experience in data handling and analysis, which supported the systematic organisation, extraction, and interpretation of the data. This combination of skills helped strengthen the overall review process. Extracted data were consolidated using descriptive statistics and qualitative content analysis.

### Risk-of-bias assessment

A risk of bias assessment was conducted to assess the quality of included publications using risk of bias tools developed by the Cochrane Collaboration: the RoB 2 tool for randomized trials [7], the RoB 2 tool for cluster randomized trials and the ROBINS-I tool for non-randomized interventional trials [8]. Risk of bias assessment was performed manually by two independent reviewers. Any discrepancies identified during the review process were first discussed among the primary reviewers with the goal of achieving consensus. When consensus was not attainable, a third reviewer provided an independent assessment to resolve the disagreement.

## Results

### Study selection

**Fig. 1:**
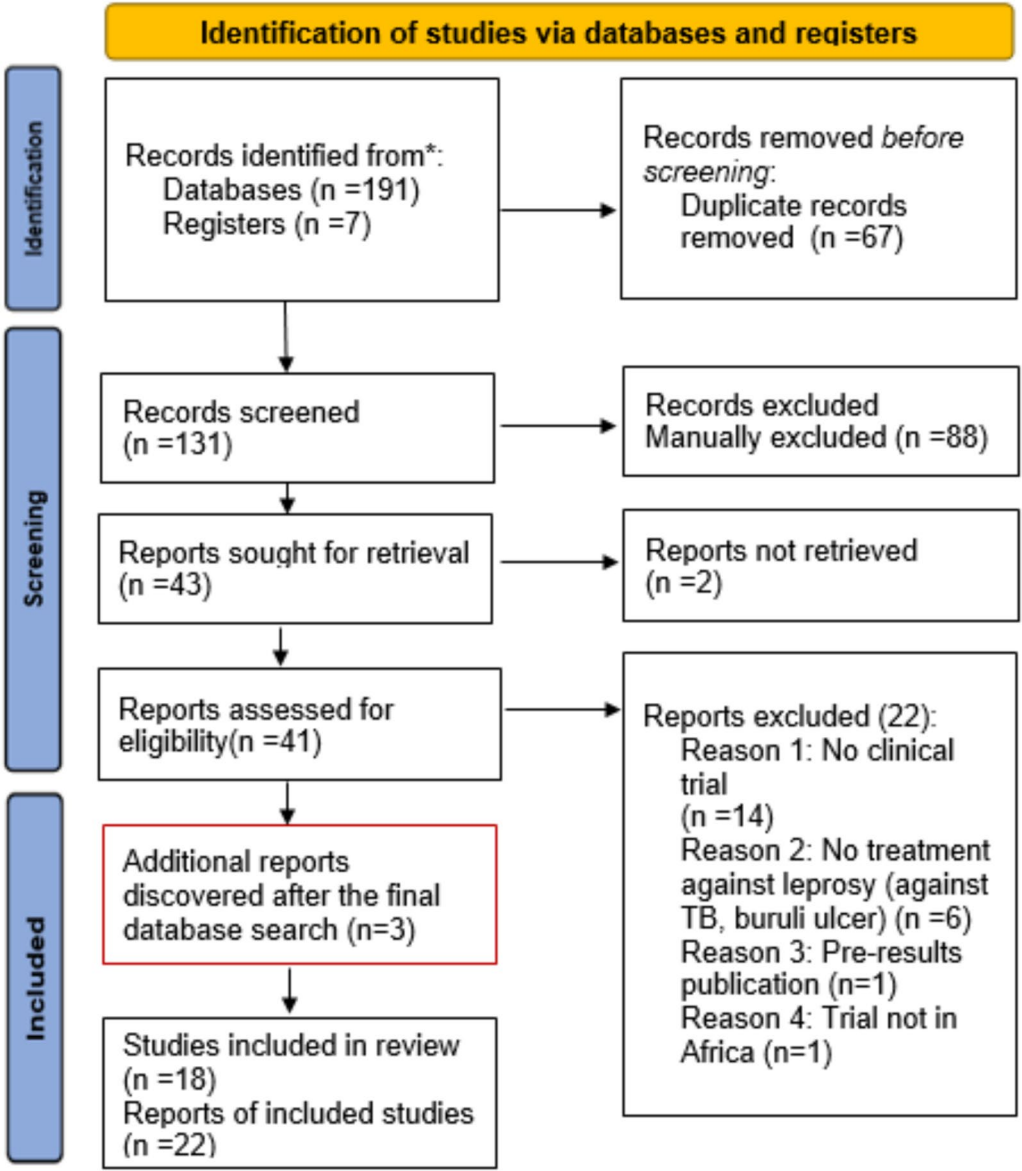
PRISMA flow diagram

A total of 198 records were identified in the above-mentioned electronic databases and registries. After removing duplicates, 131 records remained. Following title and abstract screening, 43 records were identified for eligibility assessment. Full text could not be obtained for 2, hence 41 records were fully screened for eligibility. Of these, 19 records were eligible. In addition, 3 other reports were identified and included in the final analysis. The additional 3 reports were identified in the reference section of the included publications. The finished search resulted in 22 reports of 18 clinical trials (see Fig. 1). This discrepancy in numbers arose, because several papers represented secondary analyses, follow-up investigations, or combined reporting of multiple trial phases. None of these additional publications/reports presented a duplication of previously published results. Consequently, the number of publications exceeds the number of registered trials.

### Trial characteristics

The included reports were published between 1951 and 2021, the reported trials were conducted between 1949 and 2011. Countries of conduct were Zimbabwe, Uganda, Mali, Malawi, Kenya, Ethiopia, Nigeria, Morocco and the Democratic Republic of Congo. Uganda, Ethiopia and Malawi were involved in the highest number of clinical trials.

14 clinical trials had a control group, 10 were randomized, and 6 were blinded. A clear description of the randomization process or whether randomization took place was not always available. One clinical trial that was declared randomized by the author, seemed to be pseudo-randomized based on the description given in the publication and was handled as such in this review. Pseudo-randomized trials were classified based on the description of allocation procedures. In these studies, true randomization was not used; instead, participants were assigned to one of the two study groups in a fixed, alternating sequence. A detailed overview of study characteristics is shown in Table 1.

**Table 1:** All trial characteristics

No	Authors	Title	Year of publication	Period of study conduct	Type of Trial (T/V/P/TR)	Randomization y/n/*	Controlled y/n/*	Blinded y/n/*	Recruiting countries
1	H. W. Wheate[13]	Preliminary report on sulphetrone therapy in lepromatous children	1951	1949	T	*	*	n	Uganda
2	G. Currie[14]	A clinical trial of sulforthomidine ('Fanasil' Roche) in lepromatous leprosy	1966	*	T	*	y	*	Malawi
3	J. A. Kinnear Brown, M. M. Stone, I. Sutherland, S.J. Stanley	B.C.G. vaccination of children against leprosy: first results of a trial in Uganda[15]	1966	1952–1959	V	y	y	*	Uganda
		B.C.G. vaccination of children against leprosy in Uganda: results at end of second follow-up[16]	1968	1964–1966	V	y	y	*	Uganda
		BCG vaccination of children against leprosy in Uganda: final results[17]	1981	1970–1975	V	y	y	*	Uganda
4	Y. Otsyula, C. I. Bworo, H. J. Chum[18]	Four-year experience with dapsone as prophylaxis against leprosy	1971	1963	P	n	y	*	Kenya (Buyala, Samia)
5	R. Rohde[19]	Report of combined therapy in leprosy with rifampicin and Isoprodian conducted at the Bisidimo-center, Ethiopia	1975	*	T	*	y	*	Ethiopia
6	S. R. Pattyn, M. T. Rollier, R. Rollier, E. J. Saerens, P. Dockx[20]	A Controlled Clinical Trial of Continuous and Intermittent Rifampicin Therapy During an Initial 3-Month Period in Lepromatous leprosy: Final Analysis	1975	1972–1974	T	y	y	y	Casablanca, Marokko
7	J. Manungo and J.E.P. Thomas[21]	A comparison of the incidence of type 2 reactions in lepromatous leprosy with two regimens of treatment	1982	1978–1981	TR	n	y	y	Zimbabwe
8	J. Warndorff, J. BOURLAND, S. R. Pattyn[22]	Follow-up on short-course 2-month rifampicin treatment of paucibacillary leprosy	1982	*	T	y	y	y	Burundi, Ethiopia
9	THELEP Clinical Trials Subcommittee[23]	Response to Treatment by Multidrug Regimens in the THELEP Controlled Clinical Drug Trials	1983	*	T	y	y	n	Mali, India
10	F. A. J. M. Pieters, F. Woonink, and J. Zuidema[24]	A field trial among leprosy patients in Nigeria with depot injections of Dapsone and Monoacetyldapsone	1988	1986	T	n	y	y	Nigeria
11	P.A.Orege. M.Obura. C.Okeido, P.Okuku, S.Makokha and J.Nyawaw[25]	Multidrug therapy for treatment of paucibacillary leprosy in western Kenya-preliminary communications	1990	*	T	y	y	*	Kenya
12	S. R. Pattyn, J. A. Husser, G. Baquillon, M. Maiga & P. Jamet[26]	Evaluation of five treatment regimens, using either Dapsone monotherapy or several doses of Rifampicin in the treatment of paucibacillary leprosy	1990	1981–1986	T	y	*	*	Mali
13	S.R. Pattyn, G. Groenen, L.J. Anssens, L. Kuykens, L. B. Mput[27]	A controlled therapeutic trial in paucibacillary leprosy comparing a single dose of rifampicin with a single dose of rifampicin followed by 1 year of daily dapsone. The Collaborative Study Group for the Treatment of Leprosy in Zaire	1991	1983–1985	T	y	y	*	Democratic Republic of Kongo (DRC)
14	Jorg M. Pönnighaus and Gjalt Boerrigter[28]	Are 18 doses of WHO/MDT sufficient for multibacillary leprosy; results of a trial in Malawi	1995	1987	T	y	y	*	Malawi
15	Prof Paul E M Fine, Karonga Prevention Trial Group	Randomised controlled trial of single BCG, repeated BCG, or combined BCG and killed *Mycobacterium leprae* vaccine for prevention of leprosy and tuberculosis in Malawi. Karonga Prevention Trial Group[29]	1996	1986–1989	V	y	y	y	Malawi
		BCG re-vaccination in Malawi: 30-year follow-up of a large, randomised, double-blind, placebo-controlled trial[30]	2021	1986–1989	V	y	y	y	Malawi
16	Saba M. Lambert, Shimelis D. Nigusse, Digafe T. Alembo, Stephen L. Walker, Peter G. Nicholls	Comparison of Efficacy and Safety of Ciclosporin to Prednisolone in the Treatment of Erythema Nodosum Leprosum: Two Randomised, Double-Blind, Controlled Pilot Studies in Ethiopia[31]	2016	2011–2012	TR	y	y	y	Ethiopia
		A Randomized Controlled Double-Blind Trial of Ciclosporin versus Prednisolone in the Management of Leprosy Patients with New Type 1 Reaction, in Ethiopia[32]	2016	*	TR	y	y	y	Ethiopia
17	S.N.S. Marlowe, R. Leekassa, E. Bizuneh, J. Knuutilla, P. Ale, B. Bhattarai, H. Sigdel, A. Anderson, P.G. Nicholls, A. Johnston, D. Holt, D.N.J. Lockwood	Response to ciclosporin treatment in Ethiopian and Nepali patients with severe leprosy Type 1 reactions [33]	2007	April 2001 –March 2002	TR	n	n	n	Nepal, Ethiopia
18	Roy E. Pfaltzgraff	The Control of Neuritis in Leprosy with Clofazimine [34]	1972	*	T	n	n	n	Nigeria

*no information, BCG: Bacillus Calmette–Guérin, T: Treatment, V: Vaccination, P: Prophylaxis, TR: Treatment Reactions, y: yes, n: no

The sample sizes of the clinical trials varied greatly, ranging from a treatment trial involving 25 participants to a vaccine trial involving 121,020 participants. Information on target sample size prior to the start of recruitment was not always provided.

Out of the 18 trials identified, 12 evaluated leprosy treatment (summarized in Table 2), 2 evaluated vaccines (summarized in Table 3), 1 evaluated post-exposure prophylaxis (summarized in Table 4 at the end of the section patient population), and 3 evaluated treatments of leprosy reactions (summarized in Table 5). Dapsone and rifampicin (RMP) were among the most frequently tested medications in the 12 prophylaxis and treatment trials with 10 (83%) evaluating dapsone and 8 (67%) evaluating RMP and dapsone. All vaccine trials evaluated the BCG vaccine, the prophylaxis trial evaluated dapsone and the treatment reaction trials tested clofazimine, dapsone, ciclosporin and prednisolone. These compounds were used as control or intervention.

**Table 2:** Treatment trials

No	Objective	Endpoints	Trial arms	Reached sample size	Population	How diagnosis confirmed	Sex	#Median + Mean ~ age range of recruited patients
1	*	(1) Clinical Improvement (2) Bacteriological improvement (3) Reactions	(1) Sulphetrone	25	LL children	*	Male and female	*
2	Effectiveness of sulforthomidine against leprosy	(1) Improvement of BI after 6 months (2) Incidence of ENL during the trial (6 months) (3) Reduction in Incidence of side effects after 6 months (4) Clinical Improvement after 6 months	(1) Sulforthomidine (2) Dapsone	90	LL and lepromin-negative adults	Biopsies and ear lobe smears	*	*
5	Better treatment in combined therapy and control dapsone group	(1)Percentage of BI and MI	(1) RMP and Isoprodian (2) Dapsone	80 (1) 62 (2) 18	LL and BL adults	Skin smears	Male and female	*
6	Compare the effect of daily administration of rifampicin with a once weekly administration	(1) evolution of BI and MI after 3 months (2) Incidence of ENL after 3 months (3) Evolution of weight and ESR (4)Improvement of anaesthesia of extremeties during the first 3 months	(1) RMP 450 (2) RMP 900 (3) CLO 300 (4) DDS 100	93 (1) 25 (2) 25 (3) 21 (4) 22	LL patients	Skin biopsy	Male (9(5) and female (3(4)	~ : Male (1) 14–65 (2) 16–55 (3) 16–61 (4) 20–72 Female (1) 15–62 (2) 17–53 (3) 15–50 (4) 17–75 + : Male (1) 35.8 (2) 32.3 (3) 40.4 (4) 39.0 Female (1) 38.7 (2) 25.7 (3) 42.2 (4) 42.7
8	comparison of two treatments regarding curing leprosy	(1) cure rate/clinical healing (2) cases of neuritis	(1) Dapsone (2) RMP	27 (1) Addis Ababa: 7 (2) Burundi: 9, (2) Addis Ababa: 11	PB patients	Clinically and histologically confirmed, BI = 0 or 1	*	*
9	Evaluate the effectiveness of different multidrug regimens for leprosy, specifically in the context of drug resistance	(1) proportion of persisting *M. leprae* (2) mean change of the BI per unit time (1 year) (3) clinical change after 2 years (4) change of histopathologic classification	(1) A1 (RMP, clofazimine and dapsone, each in a daily dose of 600, 100, and 100 mg, respectively in Chingleput) (2) CC (RMP, in a single initial dose of l 5 00mg, and dapsone, 100 mg daily Chingleput) (3) D1 (RMP, in a single initial dose of 1500mg, clofazimine, in a daily dose of 100 mg for the first 3 months, and dapsone, 100 mg daily Chingleput) (4)A2(RMP, prothionamide and dapsone, each in a daily dose of 600, 500, and 100 mg) (5) CB (RMP, in a single initial dose of l 5 00mg, and dapsone, 100 mg daily) (6) E2 (RMP, 900 mg once weekly, and prothionamide, 500 mg daily for the first 3 months, together with dapsone, 100 mg daily)	203 *(Mali 90)* (1) 39 (2) 38 (3) 36 (4)* 11* (5)* 43* (6)* 36*	LL adults	Skin smears	Male and female	+ : Mali persisters 27.1 non persisters 28.2 India persisters 28 non persisters 30.6
10	monitoring drug concentrations in serum and patient preferences for treatment methods, applicability of both injections in managing leprosy	(1) mean DDS concentration after 28 days (2) clearance rate of a drug	(1) DDS (2) MADDS	74 (1) 49 (2) 13	Leprosy outpatients	^*^	Male (1) 30 (2) 8 Female (1) 19 (2) 5	~ : Male (1) 28–52 (2) 38–56 Female (1) 32–48 (2) 39–51 + : Male (1) 40 (2) 47 Female (1) 40 (2) 45
11	Evaluating the efficacy, acceptability, toxicity and cost-effectiveness of the WHO recommended multidrug regimen. At the same time, The study is interested in developing an alternative regimen that could be equally effective in treatment of paucibacillary leprosy in developing countries	(1) Clinical cure (2) Toxicity (3) Compliance	(1) WHO MDT (2) Modified-MDT (RMP 1500mg repeated after 3 months with dapsone 100mg daily for 6 months)	127 (1) 64 (2) 63	BI < 2 and PB adults	Skin smears	Male 35 Female 92	~ : 0–50
12	The objective of the present study is to define short-course treatment regimens for PB leprosy and to compare them with the 'classical' dapsone treatment and the WHO–PB regimen	(1) cure rate (2) relapse rate	(1) DDS (2) RMP 8x (3) RMP 12x (4) WHO–PB: RMP + DDS (5) RMP–DDS 6d	252 (1) 33 (2) 46 (3) 40 (4) 60 (5) 73	PB patients	skin smears and skin biopsy	*	*
13	compare the efficacy of a single dose of RMP, 40 mg/kg body weight, with the previously evaluated regimen consisting of RMP 1500 mg, single dose, followed by 1 year of daily 100mg DDS	(1) Proportion of patients cured (2) Relapse rate after cure at 5 years	(1) Treatment U: single dose RMP 40mg/Kg bodyweight (2) Treatment A: treatment of RMP 1500mg, followed by 1 year of daily Dapsone 100mg	487	PB patients	Skin smears from earlobe, biopsy	Male and female	*
14	Whether treatment of MB patients can be shortened without increasing the number (percentage) of unfavorable outcomes	(1) Disabilities	(1) 18 doses MDT (2) 30 doses MDT	305 (1) 157 (2) 148	BI > = 2 and untreated MB patients	slit-skin smears and skin biopsy	Male and female	~ : 30–44
18	controlling neuritic complications of leprosy	(1) Clinical improvement nerve enlargement (2) Clinical improvement sensory loss (3) Clinical improvement motor loss	(1) Clofazimine + corticosteroids (2) Clofazimine	51	Leprosy patients	Clinical classification	Male and female	~ : 11–50

^*^no information, LL: Lepromatous Leprosy, BI: Bacterial Index, ENL: Erythema Nodosum Leprosum, BL: Borderline Leprosy, MI: Morphological Index, PB: Paucibacillary Leprosy, RMP: Rifampicin, DDS:Diaminodiphenylsulfone, also known as Dapsone, MADDS: Monoacetyldapsone, WHO: World Health Organization, MDT: Multi Drug Therapy, MB: Multibacillary Leprosy

**Table 3:** Vaccine trials

No	Objective	Endpoints	Trial arms	Reached sample size	Population	How diagnosis confirmed	Sex	#Median + Mean ~ age range of recruited patients
3	aimed to evaluate the impact of BCG vaccination on preventing leprosy in children	(1) Cases of certain Leprosy Discovered in course of First Follow-up per 1000	(1) BCG (2) no BCG	17,397 (1) 8.091 (2) 8.071	relatives and contacts of leprosy patients	Tuberculin test	Male and female	*
	The purpose is to increase the numbers of very young children under study, so as to permit in due course a more precise assessment of the efficacy of vaccination soon after birth	(1) Cases of certain Leprosy Discovered in course of second Follow-up per 1000	(1) BCG	19,096	all contacts or relatives of leprosy and all free of leprosy lesions	Tuberculin test	Male and female	*
	Discovering more about the prevention of leprosy	(1) Cases (incidence) of certain Leprosy Discovered in course of final Follow-up per 1000 (benefit) (2) Reduction of incidence	(1) BCG	17,397	children, all contacts or relatives of known leprosy patients, and all free of visible leprosy lesions	Skin smears	Male (1) 8233 Female (1) 7987	~ : 0—more than 16
15	Use and effectiveness of adding *Mycobacterium leprae* to the BCG vaccine against leprosy	(1) Incidence rate ratios	(1) No BCG scar (2) with BCG scar	121,020 (1) 66,155 (2) 54,865	Leprosy patients	Biopsy	Male and female	~ : 0 to above 60
	Effect of repeat BCG, and, therefore, on the comparison of BCG alone versus placebo in those who were scar-positive at recruitment	(1) number of leprosy events	(1) BCG (2) placebo	23,502	All individuals with no evidence of tuberculosis, leprosy or other serious disease	Leprosy examination	Male and female	*

*no information, BCG: Bacillus Calmette-Guérin

**Table 4:** Prophylaxis trial

No	Objective	Primary endpoints	Trial arms	Reached sample size	Population	How diagnosis confirmed	Sex	#Median + Mean ~ age range of recruited patients
4	Whether dapsone is an effective prophylactic agent against leprosy	(1) incidence rate for 3 years	(1) Dapsone 2) No treatment	4773	Children with leprosy	*	Male and female	school years 1–4 ~

*no information

**Table 5:** Treatment reaction trials

No	Objective	Endpoints	Trial arms	Reached sample size	Population	How diagnosis confirmed	Sex	#Median + Mean ~ age range of recruited patients
7	Comparison of the incidence of type 2 reactions in lepromatous leprosy with a clofazimine/dapsone regimen and an HT3/dapsone regimen	(1) Incidence of reactions (2) Reactions related to dose of dapsone (3) Reactions related to age (4) Reactions related to sex (5) Reactions related to country of origin (6) Reactions related to duration of disease (7) Reactions related to the duration of treatment	(1) Clofazimine and Dapsone (2) HT3 and Dapsone	54 (1) 27 (2) 27	adult LL patients	Ear lobe scraping, clinical observation	Male and female	*
16	Comparing the efficacy and side effect profile of ciclosporin + prednisolone against prednisolone alone in the treatment of patients with either new ENL or chronic and recurrent ENL	(1) Mean number ENL recurrence episodes	(1) Ciclosporin and Prednisolone (2) Prednisolone	33	Individuals with new acute ENL or chronic ENL	patients with leprosy had crops of tender subcutaneous skin lesions	Male and female	#: new ENL Ciclosporin: 30 Prednisolone: 30 chronic ENL Ciclosporin: 27 Prednisolone: 30 ~ : 18–65
	*	(1) change in severity at 28 weeks (2) change in motor function and sensory loss	(1) Ciclosporin and Prednisolone (2) Prednisolone alone	73 (1) 35 (2) 38	adult and children (above 30kg) with new and recent onset T1R	skin smears	Male and female	#: Ciclosporin: 27 Prednisolone: 34 ~ : 18–65 years
17	Comparing the effectiveness of 12-week course of prednisolone only (different studies) to ciclosporin treated patients	(1) Nerve pain and tenderness (2) Nerve function impairment (3) Relapse of T1Rs	(1) Ciclosporin (5mg/kg/day) and 40mg prednisolone for 5 days, then only ciclosporin for 12 weeks	43 (33 in Ethiopia, 10 in Nepal)	Adults with acute severe T1R	skin lesions	Male and Female	~ : 18–65 years + : 34 in Ethiopia 36 in Nepal

*no information, LL: Lepromatous Leprosy, HT3: Isoniazid 300 mg plus Thioacetazone 150 mg, ENL: *Erythema nodosum leprosum*, T1R: Type 1 reaction

**Fig. 2:**
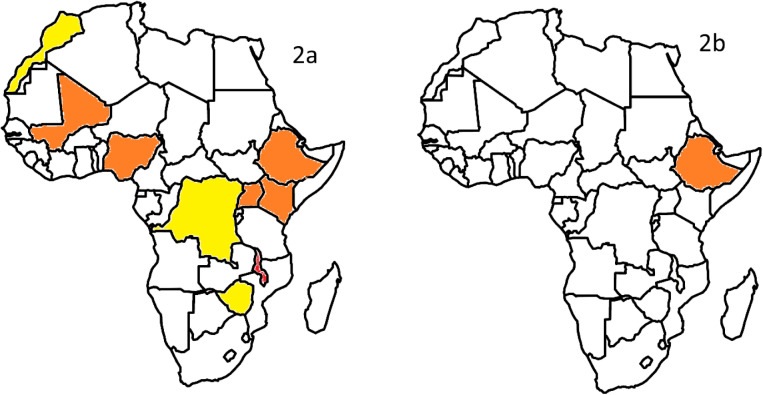
Clinical trial conduct in African countries. 2a shows countries involved in clinical trial conduct before the year 2000, 2b shows countries involved in clinical trial conduct after the year 2000. None of the trials started recruitment in 2000. Legend: yellow: 1 trial, orange: 2 trials, red: 4 trials

Out of the 18 trials, 15 were published before and 2 after the year 2000. One trial was reported in two separate publications spanning both time periods, with one being published before and one after 2000. Figure 2 shows the African map with the number of trials per country that started recruitment before and after the year 2000.

**Fig. 3:**
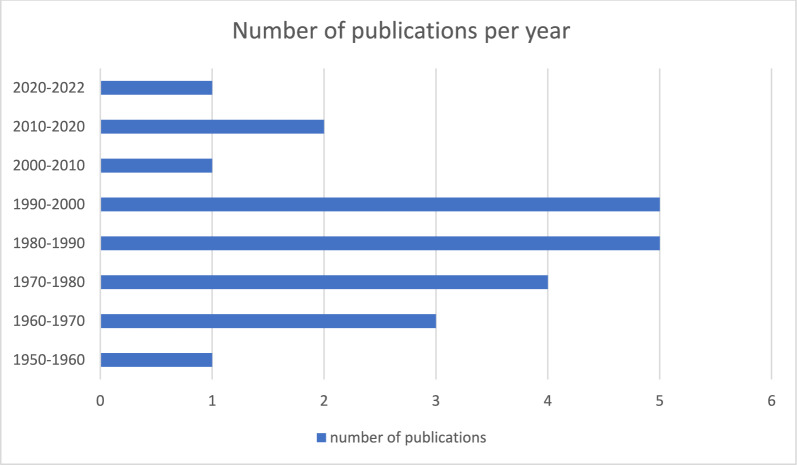
Publications with clinical trial data from Africa

Complementary to Figs. 2, 3 shows the number of publications per decade.

The study durations ranged from 8 weeks to 30 years. Outcomes varied across trials and indications and included clinical and bacteriological improvement, cure rate and incidence, clearance rate, toxicity and compliance, relapse rate, disabilities and changes in severity.

### Patient populations

Some trials were restricted to children or adults only. Out of the 18 trials, 4 only recruited children, 5 only adults. More specific descriptions of age requirements beyond that were not described. 9 trials recruited participants without age restrictions. Other eligibility criteria included history or presence of symptoms of leprosy, or the exact opposite, i.e., having no signs of leprosy. 6 clinical trials recruited only patients with Lepromatous Leprosy (LL), 4 specifically patients with Paucibacillary Leprosy (PB), and one included Borderline Leprosy (BL) patients, multibacillary patients only, respectively. 5 trials did not specify the leprosy type and one trial recruited people with no signs of leprosy. Leprosy patients were diagnosed with skin smears and skin biopsies.

### Quality of included studies

As part of this work, a risk of bias assessment was conducted for all publications and their mentioned outcomes. 9 records presented data from individually randomized trials, 2 presented data from cluster randomized trials and 7 trials were not adequately randomized. For the individually randomized trial records, 23 outcomes were presented and assessed for risk of bias. It was not made clear whether the outcomes were primary or secondary (Table 6).

**Table 6:** RoB 2 tool for publications of randomized trials

No	Publication Title	Outcome	Overall bias
6	A Controlled Clinical Trial of Continuous and Intermittent Rifampicin Therapy During an Initial 3-Month Period in Lepromatous leprosy: Final Analysis	Evolution of BI and MI after 3 months	High risk
		Incidence of ENL after 3 months	
		Evolution of weight and ESR	
		Improvement of anaesthesia of extremities during the first 3 months	
8	Follow-up on short-course 2-month rifampicin treatment of paucibacillary leprosy	Cure rate/clinical healing	High risk
		Cases of neuritis	
9	Response to Treatment by Multidrug Regimens in the THELEP Controlled Clinical Drug Trials	Proportion of persisting *M. leprae*	High risk
		Mean change of the BI per unit time (1 year)	
		Clinical change after 2 years	
		Change of histopathologic classification	
11	Multidrug therapy for treatment of paucibacillary leprosy in western Kenya–preliminary communications	Clinical cure	High risk
		Toxicity	
		Compliance	
12	Evaluation of five treatment regimens, using either Dapsone monotherapy or several doses of Rifampicin in the treatment of paucibacillary leprosy	Cure rate	High risk
		Relapse rate	
13	A controlled therapeutic trial in paucibacillary leprosy comparing a single dose of rifampicin with a single dose of rifampicin followed by 1 year of daily dapsone. The Collaborative Study Group for the Treatment of Leprosy in Zaire	Proportion of patients cured	Some concerns
		Relapse rate after cure at 5 years	
14	Are 18 doses of WHO/MDT sufficient for multibacillary leprosy; results of a trial in Malawi	Disabilities	High risk
15	BCG re-vaccination in Malawi: 30-year follow-up of a large, randomised, double-blind, placebo-controlled trial	Number of leprosy events	High risk
15	Randomised controlled trial of single BCG, repeated BCG, or combined BCG and killed *Mycobacterium leprae* vaccine for prevention of leprosy and tuberculosis in Malawi. Karonga Prevention Trial Group	Incidence rate ratios	Some concerns
16	A Randomized Controlled double-blind Trial of Ciclosporin versus Prednisolone in the Management of Leprosy Patients with New Type 1 Reaction, in Ethiopia	Change in severity at 28 weeks	Some concerns
		Change in motor function and sensory loss	
16	Comparison of Efficacy and Safety of Ciclosporin to Prednisolone in the Treatment of Erythema Nodosum Leprosum: Two Randomised, Double-Blind, Controlled Pilot Studies in Ethiopia	Mean number ENL recurrence episodes	Some concerns

ENL: *Erythema Nodosum Leprosum*, BI: Bacterial lndex, MI: Morphological lndex, ESR: Erythrocyte Sedimentation Rate

In addition to the tables below, a heatmap was created for each table to improve visual clarity. (See Supplementary file 4).

The RoB 2 tool for cluster randomized trials showed a high risk of bias for both eligible trials, as demonstrated in Table 7.

**Table 7:** RoB 2 tool for publications of cluster randomized trials

No	Publication title	Outcome	Overall bias
4	Four-year experience with dapsone as prophylaxis against leprosy	Incidence rate for 3 years	High risk
10	A field trial among leprosy patients in Nigeria with depot injections of Dapsone and Monoacetyldapsone	Mean DDS concentration after 28 days	High risk
		Clearance rate of a drug	

DDS: Diaminodiphenylsulfone

The ROBINS-I tool concluded on 3 out of 7 trials to be moderate, 2 serious and 2 without information on the risk of bias. 25 outcomes were presented and assessed for risk of bias. Further details are shown in Table 8.

**Table 8:** ROBINS-I tool for publications of non-randomized trials

No	Publication title	Outcome	Overall bias
1	Preliminary report on sulphetrone therapy in lepromatous children	Clinical improvement	No information
		Bacteriological improvement	
		Reactions	
2	A clinical trial of sulforthomidine ('Fanasil' Roche) in lepromatous leprosy	Improvement of BI after 6 months	Serious
		Incidence of ENL during the trial (6 months)	
		Reduction in incidence of side effects after 6 months	
		Clinical improvement after 6 months	
3	B.C.G. vaccination of children against leprosy: first results of a trial in Uganda	Cases of leprosy discovered in course of first follow-up per 1000	Moderate
3	B.C.G. vaccination of children against leprosy in Uganda: results at end of second follow-up	Cases of certain leprosy discovered in course of second follow-up per 1000	Moderate
3	BCG vaccination of children against leprosy in Uganda: final results	Cases (incidence) of certain leprosy discovered in course of final follow-up per 1000 (benefit)	Moderate
		Reduction of incidence	
5	Report of combined therapy in leprosy with rifampicin and Isoprodian conducted at the Bisidimo-center, Ethiopia	Percentage of BI and MI	No information
7	A comparison of the incidence of type 2 reactions in lepromatous leprosy with two regimens of treatment	Incidence of reactions	Moderate
		Reactions related to dose of dapsone	
		Reactions related to age	
		Reactions related to sex	
		Reactions related to country of origin	
		Reactions related to duration of disease	
		Reactions related to the duration of treatment	
17	Response to ciclosporin treatment in Ethiopian and Nepali patients with severe leprosy Type 1 reactions	Nerve pain and tenderness	Moderate
		Nerve function	
		Relapse of T1Rs	
18	The Control of Neuritis in Leprosy with Clofazimine	Clinical improvement nerve enlargement	Serious
		Clinical improvement sensory loss	
		Clinical improvement motor loss	

MI: Morphological lndex, BI: Bacterial lndex, ENL: Erythema Nodosum Leprosum

## Conclusions and discussion

This scoping review provides an overview of the landscape of clinical trials evaluating medical interventions in the field of leprosy conducted in Africa.

Despite the ongoing burden of leprosy on the continent, this review demonstrates a notable scarcity of clinical trials that specifically focuses on the African patient population. Interestingly, most clinical trials were conducted in Malawi and Ethiopia (Table 1). To date, Ethiopia remains a key location for leprosy care and research in Africa.

A review of clinical trials listed in the WHO International Clinical Trial Platform that started after 2022 shows two new interventional trials registered for 2024 and 2025. One of them is being conducted in Egypt, while the other is taking place in Mali.

For informational purposes, illustrative contextual observations were made at a later stage to highlight the scarcity of trials conducted. A rapid, non-systematic search of the PubMed database without geographical restrictions yielded a total of 723 clinical trial publications in the field of leprosy. Conversely, the same search, but with the additional filter "Africa", resulted in a total of 44 publications. A further comparison of search results showed that replacing the geographic filter “Africa” with “Asia” yielded 115 publications, suggesting a markedly higher research output from the Asian continent.

As an additional contextual comparison with another neglected tropical disease (NTD), schistosomiasis, a rapid, non-systematic search identified 377 publications on the search conducted in Africa. In detail, 277 publications were identified in PubMed, 53 are registered in the WHO ICTRP and 47 of which were found in the Cochrane database. A search of the Infolep database was not performed, as it is primarily intended for leprosy research. In comparison, the leprosy PubMed search identified 49 publications, 0 registered in WHO ICTRP, 42 found in the Cochrane database and 107 publications identified in the Infolep database. No duplicate removal, formal screening, or comparative analysis was performed. Nevertheless, this overview suggests that the volume of schistosomiasis-related research activity in Africa importantly exceeds that of leprosy, thereby indicating disparities in research attention between NTDs on the continent.

With the global elimination target achieved, leprosy was declared eliminated as a public health problem by the WHO in 2000. As opposed to an epidemiological elimination target, this target was defined by a threshold of less than 1 case per 10,000 population, acknowledging continued transmission [9]. Despite persistence of leprosy cases, declining research attention followed which is reflected by the findings of this review showing 82% of clinical trial data being published before the year 2000. The dominance of clinical trials conducted before 2000 might limit the direct policy relevance of the current evidence base. Many of these studies were conducted under different health systems, diagnostics and treatment protocols, making their findings only partly applicable to today’s context. While they provide important historical insight, the lack of recent research highlights a gap that constrains evidence-based decision-making for current leprosy control.

The WHO acknowledged the need to aim for zero leprosy in the current Global Leprosy Strategy and defined 4 pillars to achieve this ambitious objective. The development of more effective treatments for leprosy and leprosy reactions, preventive vaccines and post-exposure prophylaxis support these goals by contributing to reduced transmission, managing leprosy and its complications and preventing new disability [10, 11]. For research studies and resulting interventions to be effective, patient populations participating in clinical trials should be representative of the patient populations that ultimately benefit from their results, as epidemiological, social or biological differences between populations can impact the efficacy and effectiveness of a medical intervention. To successfully put this into practice, collaborations need to be fostered, and research capacities need to be built. Furthermore, to facilitate the conduct of additional studies and reinforce existing research centers, strategies could be implemented to secure long-term, locally governed funding, enhance infrastructures for clinical trials, and expand training programs to facilitate clinical research.

### Limitations

In several publications, particularly from before the turn of the millennium, descriptions about the planning and design of the clinical trials lacked details. This lack of detail might be due to historical differences of reporting standards and potentially the absence of a widely accepted and formalized publication standard, such as the Consolidated Standards of Reporting Trials (CONSORT) statement, which was first introduced in 1996 [12]. This should also be taken into consideration when interpreting the high risk of bias ratings in publications published before 2000 as they may reflect historical reporting standards rather than necessarily poor study conduct.

It is also important to acknowledge that the publications captured in this review may not be fully comprehensive. Despite broad search strategies, some relevant studies may not have been identified due to the choice of electronic resources, limitations in database indexing or inconsistent terminology. These factors may have affected the completeness and balance of the evidence mapped in this scoping review.

### Main conclusion

Evidence-based treatment and prophylaxis are basic requirements for successful disease prevention and control. The scarcity of published clinical trial data from African countries limits the ability to translate clinical trial findings to the African patient population, potentially hindering the effectiveness of an in principle efficacious intervention. To address these gaps, future research efforts should prioritize the inclusion of the African patient population in leprosy clinical trials and build clinical trial capacities alongside. This can contribute to more effective and context appropriate interventions that help progress towards leprosy elimination in Africa.

## Supplementary Information

Additional file 1.

Additional file 2.

Additional file 3.

Additional file 4.

## Data Availability

Data sharing is not applicable to this article as no data sets were generated or analysed during the current study.

## Abbreviations

BCG: Bacillus Calmette-Guérin
BI: Bacterial index
BL: Borderline leprosy
BT: Borderline tuberculoid
DDS: Diaminodiphenylsulfone, also known as Dapsone
ENL: Erythema nodosum leprosum
ESR: Erythrocyte sedimentation rate
HT3: Isoniazid 300mg plus thioacetazone 150mg
LL: Lepromatous leprosy
MADDS: Monoacetyldapsone
MB: Multibacillary leprosy
MDT: Multi drug therapy
MI: Morphological index
PB: Paucibacillary leprosy
RMP: Rifampicin
T1R: Type 1 reaction
TT: Tuberculoid leprosy
WHO: World Health Organization
