# Supplementary material for: Involvement of African patient populations in clinical trials on leprosy: a scoping review
Source: Trop Med Health. 2026 Apr 3;54:69. doi: 10.1186/s41182-026-00913-x (PMC13077914; doi:10.1186/s41182-026-00913-x)
Supplement: Supplementary file 4 — Additional file 4. [file 41182_2026_913_MOESM4_ESM.pdf]

Risk of bias: ROB-2

Study No.	Outcome	D1	D2	D3	D4	D5	Overall
6	evolution of BI and MI after 3 months	!	!	+	!	!	-
6	Incidence of ENL after 3 months	!	!	+	-	!	-
6	Evolution of weight and ESR	!	!	+	+	!	-
6	Improvement of anaesthesia of extremeties during the first 3 months	!	!	+	-	!	-
8	cure rate/clinical healing	!	!	+	-	!	-
8	cases of neuritis	!	!	+	-	!	-
9	mean change of the BI per unit time (1 year)	-	!	-	-	!	-
9	clinical change after 2 years	-	!	-	-	!	-
9	change of histopathologic classification	-	!	-	-	!	-
9	proportion of persisting M. leprae	-	!	-	-	!	-
11	toxicity	!	!	-	-	!	-
11	compliance	!	!	-	+	!	-
11	clinical cure	!	!	-	+	!	-
12	cure rate	+	!	-	-	!	-
12	relapse rate	+	!	-	-	!	-
13	relapse rate after cure at 5 years	!	!	+	!	!	!
13	proportion of patients cured	!	!	+	!	!	!
14	disabilities	+	!	+	-	!	-
15	number of leprosy events	+	+	+	-	!	-
15	Incidence rate ratios	+	+	+	+	!	!
16	change in severity at 28 weeks	+	+	+	!	!	!
16	change in motor function and sensory loss	+	+	+	!	!	!
16	mean number ENL recurrence episodes	+	+	+	!	!	!

- High risk ! Some concerns + Low risk

D1 Randomisation process
D2 Deviations from the intended interventions
D3 Missing outcome data
D4 Measurement of the outcome
D5 Selection of the reported result

Risk of bias: ROB-2 cluster

Study ID	Outcome	Weight	D1a	D1b	D2	D3	D4	D5	Overall
4	incidence rate for 3 years	1	!	!	!	+	-	!	
10	mean DDS concentration after 28 days	1	!	!	+	-	+	!	
10	clearance rate of a drug	1	!	!	+	-	+	!	

+ Low risk

! Some concerns

- High risk

D1a Randomisation process

D1b Timing of identification or recruitment of participants

D2 Deviations from the intended interventions

D3 Missing outcome data

D4 Measurement of the outcome

D5 Selection of the reported result

Risk of bias: ROBINS-I

Study No.	Outcome	D1	D2	D3	D4	D5	D6	D7	Overall
1	Clinical Improvement		+	+			!		
1	Bacteriological improvement		+	+			!		
1	Reactions		+	+			!		
2	Improvement of BI after 6 months	-	+	+	+	!	-		-
2	Incidence of ENL during the trial (6 months)	-	+	+	+	!	-		-
2	Reduction in Incidence of side effects after 6 months	-	+	+	+	!	-		-
2	Clinical Improvement after 6 months	-	+	+	+	!	-		-
3	Cases of Leprosy Discovered in course of First Follow-up per 1000	+	+	+	+	!	+		!
3	Cases of certain Leprosy Discovered in course of second Follow-up per 1000	+	+	+	+	!	!		!
3	Cases (incidence) of certain Leprosy Discovered in course of final Follow-up per 1000 (benefit)	+	+	+	+	!	+		!
3	Reduction of incidence	+	+	+	+	!	+		!
5	Percentage of BI and MI		+	!			!		
7	Incidence of reactions	-		+	+	+	!		!
7	Reactions related to dose of dapsone	-		+	+	+	!		!
7	Reactions related to age	-		+	+	+	!		!
7	Reactions related to sex	-		+	+	+	!		!
7	Reactions related to country of origin	-		+	+	+	!		!
7	Reactions related to duration of disease	-		+	+	+	!		!
7	Reactions related to the duration of treatment	-		+	+	+	!		!
18	Clinical improvement nerve enlargement		!	-	-	+	!		-
18	Clinical improvement sensory loss		!	-	-	+	!		-
18	Clinical improvement motor loss		!	-	-	+	!		-

- Critical
! Moderate
+ Low
- Serious
No information

D1 Bias due to confounding
D2 Bias in selection of participants into the study
D3 Bias in classification of interventions
D4 Bias due to deviations from intended interventions
D5 Bias due to missing data
D6 Bias in measurement of outcomes
D7 Bias in selection of the reported result
